# Association Between COVID19 Vaccination Uptake and Socio-Demographic Characteristics Among Pregnant Women in Kenya

**DOI:** 10.24248/eahrj.v9i1.819

**Published:** 2025-09-30

**Authors:** Sylvia Ayieko, Sarah E. Messiah, Kimberly Baker, Christine Markham

**Affiliations:** a Department of Community and Behavioral Health, The University of Iowa, College of Public Health, Iowa City, IA 52242 USA; b Peter O'Donnell Jr. School of Public Health, UT Southwestern Medical Center: Dallas, TX 75390, USA; c Department of Health Promotion and Behavioral Sciences, The University of Texas Health Science Center at Houston, School of Public Health, Houston, TX 77030, USA.

## Abstract

**Background:**

Vaccination is considered more cost-effective in controlling the spread of COVID19 compared to other preventative measures. Pregnant women infected with COVID19 were likely to have severe COVID19 complications compared to uninfected women. This study examined the relationship between COVID19 vaccination uptake and socio-demographic characteristics among pregnant women in Kenya.

**Methods:**

We conducted a secondary analysis using data from a pilot study examining COVID19 vaccination uptake among pregnant women in Kenya. We used descriptive analysis to report the proportions and chi 2 to assess if there are any significant differences between groups. Our primary outcome was COVID19 vaccination uptake. We performed logistic regressions.

**Results:**

The predominant age groups of study participants were between 25–29 years (45.1%) and 30–39 years (45.1%). The majority of the pregnant had received COVID19 vaccination, with 87% having completed the full vaccine dosage. There was a higher uptake of adenovirus vector vaccines compared to mRNA COVID19 vaccines. Pregnant women between 30–39 years were 3.81 times more likely to have received COVID19 vaccination compared to those 25–29 years (aOR: 3.81; 95% CI: 1.28–11.39, *P* =.017). After adjustment, workplace requirements for COVID-19 vaccination were associated with increased odds of vaccination (aOR: 4.65; 95% CI 1.32–16.42). The study did not show any significant relationship between comorbid conditions during pregnancy and COVID19 vaccination

**Discussion:**

The variations of uptake by age-group cohorts, education levels, and vaccine workplace requirements suggest a need for further and more robust research on COVID19 vaccination among pregnant women. While workplace vaccination requirements may have prompted COVID19 vaccination among pregnant women in this sample, the results cannot be generalized to the general population. However, our findings underscore the importance of effective public health policies at the institutional, local and national levels.

## BACKGROUND

In 2020, the World Health Organization declared coronavirus disease 2019 (COVID19) a global pandemic after several countries reported thousands of deaths and overburdened health systems due to COVID19 hospitalizations.^1–3^ Although the epidemiology of COVID19 in Africa was uncertain due to inadequate surveillance systems,^4^ available data showed that Sub-Saharan African countries reported much lower rates of COVID19 cases and mortalities compared to countries in Europe and the Americas.^3,5,6^ Research suggested that viral variants would continue emerging until the spread of COVID-19 was controlled.^7^

COVID-19 directly and indirectly impacted maternal health outcomes.^8,9^ Evidence suggested that COVID-19 infections exacerbated severe morbidities and mortality during pregnancy.^10^ Studies among countries in Sub-Saharan Africa indicated that hospitalized pregnant women with COVID19 had a higher risk of intensive care unit (ICU) admissions and deaths compared to uninfected, hospitalized pregnant women.^11–13^ Pregnant women in Kenya had a higher likelihood of COVID19 infections compared to other women of reproductive age,^14^ given that females accounted for 44% of all confirmed COVID19 cases reported in Kenya by June 2023.^15^

The high rates of COVID19 infections, complications and deaths^2,3^ prompted public health organizations, international health agencies, and governments to recommend COVID19 vaccination as a feasible strategy to mitigate the spread of the virus worldwide.^16–18^ The mass production and public distribution of COVID19 vaccines by November 2020 were among the most effective strategies to counter the high disease burden worldwide.^19^

Vaccination of entire populations is encouraged to achieve herd immunity; thus, researchers reported that vaccination against the COVID19 led to substantial reductions in COVID19 complications and deaths.^20^ COVID19 vaccination initial efforts primarily targeted healthcare providers and vulnerable populations, including pregnant women, because of the increased risk of COVID19 complications faced by these groups.^21,22^ In Sub-Saharan Africa, initial challenges faced by countries included fragile public health systems, lack of political commitment, economic challenges and low perceptions of COVID19 susceptibility.^23,24^

By joining the COVID19 Vaccines Global Access Facility (COVAX),^25^ the Kenyan government, through the Ministry of Health (MOH), planned to vaccinate at least 70% of its adult population by June 2022.^15^ However, by November 26, 2022, less than 37% of adults had received the complete dosage of the COVID19 vaccine.^3,15,26^ The Kenya Obstetric and Gynecological Society (KOGS) recommended COVID19 vaccination for pregnant women in Kenya^27^ following evidence of vaccine safety and efficacy among pregnant women in the United Kingdom.^28^ Despite the risk among pregnant women and the availability of COVID19 vaccines in Kenya, it was unclear if pregnant women in Kenya were willing to receive COVID19 vaccinations.

It was critical to investigate the uptake of COVID19 vaccines, especially as a new vaccine, given that the World Health Organization included vaccine hesitancy among the top ten global health threats.^19,29^ Various studies examining COVID19 vaccination acceptance and hesitancy among general populations reported age, education, and gender as significant predictors of COVID19 vaccination.^30,31^ Additional determinants that impact the general population include workplace requirements, region, medical comorbidities, and prior COVID19 infection.^32,33^ While similar factors may also impact COVID19 vaccination behaviours among pregnant women in Kenya, it was not certain whether other factors specific to pregnant women, such as compliance with other recommended vaccines during pregnancy and comorbidities,^34,35^ could also impact COVID19 vaccination among pregnant women in Kenya.

Considering the morbidity and mortality associated with COVID19 and the disproportionately high COVID19 disease burden among pregnant women in Kenya,^14^ evaluating factors related to COVID19 vaccination in this population was pivotal. Understanding the predictors of vaccination among pregnant women may help facilitate the implementation of more robust strategies designed to increase COVID19 vaccination and reduce the impact of future health emergencies in this population. This study aimed to determine the prevalence of COVID19 vaccination uptake and associated socio-demographic characteristics among pregnant women seeking care at two national referral hospitals in Kenya.

## METHODS

### Study Design, Setting and Participants

This secondary analysis used cross-sectional data from a pilot study on COVID19 vaccination among pregnant women in Kenya.^36^ The study was conducted in two referral hospitals in Nairobi and Uasin Gishu counties between May 2022 and October 2022 to determine COVID19 vaccination uptake, associated factors, and potential predictors of vaccine uptake. Data collected were self-reported using an electronic questionnaire delivered via Qualtrics.^37^ The survey, administered in English, primarily used questions from the National Immunization Survey-Adult COVID Module (NIS-ACM) and the Omnibus survey,^38^ but adapted a few questions relevant to pregnant women in Kenya. Study participants had to meet the following inclusion criteria: (1) be between 18 and 49 years old, (2) report being pregnant, (3) have access to a mobile device with internet access and WhatsApp application. Individuals who did not provide informed consent were excluded from the study. While some health services are provided to mature emancipated minors, we excluded women younger than 18 years because of the unclear policy guidelines for COVID19 vaccination among children at the time of data collection. Additional study procedures are outlined in the pilot study.^36^

### Ethical Considerations

Ethical clearance was waived for this specific study since it utilized secondary de-identified data. Detailed information about ethical approval for the primary study is available elsewhere.^36^

### Measures

This study examined the relationship between socio-demographic characteristics and COVID19 vaccination among pregnant women in Kenya. The study also reported the prevalence of COVID19 vaccination by vaccine brand. The dependent variable (COVID19 vaccination status) and the independent variables (socio-demographic characteristics) are described below.

**Primary Outcome Variable:** The dependent variable for this study was COVID19 vaccination uptake. In response to the question “Have you received COVID19 vaccination?”, the responses were coded as a binary outcome, where “No” was categorized as “Unvaccinated,” while “Yes” was classified as the “Vaccinated” outcome. Participants who reported receiving COVID19 vaccination were asked about the date of vaccination and the brand of vaccine they received as either “Pfizer,” “Moderna,” “AstraZeneca,” “Johnson & Johnson,” or “Other.”

**Exposure Variables:** The primary independent variables included age, education level, insurance status, disability, region, previous COVID19 infection, workplace requirements, and chronic conditions. Age was categorized as 18–24, 25–29, 30–39, or 40–49. The highest level of education attained was categorized as “Did not go to school,” “Primary,” “Secondary,” or “College.” Insurance status was categorized as “Insured” or “Uninsured.” Region or place of residence was recategorized as either “Nairobi” or “Uasin Gishu” (other surrounding counties were grouped into these two regions). Disability was categorized as either “Yes” or “No”. Previous COVID19 infection was categorized as either “Yes” or “No”; workplace requirements for COVID19 vaccination as “Yes,” “No,” or “Unemployed”; receipt of vaccines other than COVID19 vaccines as “Yes” or “No”; and chronic condition as either “Yes” or “No” (CDC, 2022).

### Statistical Analysis

Descriptive analysis was used to describe socio-demographic characteristics and the prevalence of COVID19 vaccination in this study sample. Categorical and binary variables were reported as frequencies with proportions. The differences in prevalence rates between vaccinated and unvaccinated participants were tested for significance using the Chi-square test. Chi-square tests examined the differences in prevalence.

Bivariate logistic regressions analyses were conducted to assess the odds of COVID19 vaccination uptake by each of the socio-demographic variables (age, education level, region, insurance status, disability, chronic condition, previous COVID19 infection, workplace vaccine requirements and receipt of vaccines other than COVID19 vaccines). Results were reported as odds ratios and 95% confidence intervals. In addition, multivariable logistic regressions were conducted to generate the odds and 95% confidence intervals between COVID19 vaccination uptake and the socio-demographic variables. We assumed that each independent variable selected for inclusion in the model was at least interval scaled. To identify the unique groups, we assigned dummy variables at interval levels for nominal and discrete variables such as region and workplace vaccine requirements. Statistical analyses were performed using Stata SE 18.^39^ All tests were two-sided, with *P* values less than .05 considered statistically significant.

## RESULTS

### Sample Characteristics and Descriptive Statistics

The characteristics of vaccinated and unvaccinated pregnant women in the study sample are presented in Table 1. A total of 115 participants (59 from Nairobi and 56 from Uasin Gishu) completed the survey. The predominant groups were participants between 25–29 years (39.1%) and 30–39 years (39.1%), compared to the 40 years or older group (5%). More than 67% of respondents from both regions had either attended college or university.

**TABLE 1:** Sociodemographic Characteristics of the Pregnant Study Participants (N=115)

Variable	Overall n (%) 115 (100)	Unvaccinatedn (%) 31 (27.0)	Vaccinatedn (%) 84 (73.0)	*P value*
Age				.091
18–24 years	19 (16.5)	4 (21.1)	15 (78.9)	
25–29 years	45 (39.1)	18 (40.0)	27 (60.0)	
30–39 years	45 (39.1)	8 (17.8)	37 (82.2)	
40–49 years	6 (5.2)	1 (16.7)	5 (83.3)	
Education level				.290
Primary school	3 (2.6)	2 (66.7)	1 (33.3)	
Secondary school	34 (29.6)	9 (26.5)	25 (73.5)	
College/University	78 (67.8)	20 (25.6)	58 (74.4)	
Region				.704
Nairobi	59 (51.3)	15 (25.4)	44 (74.6)	
Uasin Gishu	56 (48.7)	16 (28.6)	40 (71.4)	
Insurance status				
No	30 (26.1)	9 (30.0)	21 (70.0)	.662
Yes	85 (73.9)	22 (25.9)	63 (74.1)	
Disability				
No	100 (87.0)	27 (27.0)	21 (70.0)	.979
Yes	15 (13.0)	4 (26.7)	63 (74.1)	
Comorbid condition				
No	94 (81.7)	27 (28.7)	67 (71.3)	.366
Yes	21 (18.3)	4 (19.0)	17 (81.0)	
Previous COVID 19 infection				.689
No	67 (58.3)	19 (28.4)	48 (71.6)	
Yes	48 (41.7)	12 (25.0)	36 (75.0)	
Workplace vaccine requirements				.079
No	32 (28.6)	11 (34.4)	21 (65.6)	
Yes	51 (45.5)	8 (15.7)	43 (84.3)	
Unemployed	29 (25.9)	10 (34.5)	19 (65.5)	
Health Care Worker				.034
No	104 (90.4)	31 (29.8)	73 (70.2)	
Yes	11 (9.6)	0 (0.0)	11 (100)	
Receipt of vaccines other than COVID19				.941
No	65 (58.0)	17 (26.2)	48 (73.8)	
Yes	47 (42.0)	12 (25.5)	35 (74.5)	
Wearing a Mask				.533
No	28 (25.0)	6 (21.4)	22 (78.6)	
Yes	84 (75.0)	23 (27.4)	61 (72.6)	

**TABLE 2:** Prevalence of COVID19 Vaccination by Region and Brand

Vaccination	Status Nairobi n (%)	Uasin Gishu n (%)
Unvaccinated	15 (25.4)	16 (28.6)
Vaccinated	44 (74.5)	40 (71.4)
Vaccine Brand		
AstraZeneca	22 (50.0)	19 (47.5)
Johnson & Johnson	9 (20.4)	11 (27.5)
Modema	8 (14.8)	3 (7.5)
Pfeizer	4 (9.1)	5 (12.5)
Other/Not sure	1 (23)	2 (5.0)

Most participants (73%) reported that they had received at least one dose of a COVID19 vaccine during the study. Vaccination rates between the two regions were similar, although Nairobi had a slightly higher rate of 74.5%. There was no significant difference in the distribution by age, education, insurance status, or region among those who received COVID19 vaccines and those who had not yet been vaccinated against COVID19. All the pregnant women who were healthcare workers (nurses, social workers, or employees in a health facility) were vaccinated, and the difference between the non-healthcare workers was statistically significant (Table 1).

AstraZeneca was the most reported COVID19 vaccine, with at least 48% of participants from each region stating they received at least one dose. About a quarter of vaccinated study participants (23.8%) reported receiving the Johnson & Johnson COVID19 vaccine. The messenger RNA (mRNA) vaccines (Pfizer and Moderna) were not as frequently reported as the adenovirus vector vaccines (AstraZeneca and Johnson & Johnson). About 87% of pregnant women were fully vaccinated against COVID19, having received one dose of the Johnson & Johnson vaccine and two or more doses of the other vaccines.

Overall, COVID19 vaccination uptake was the lowest among 25–29 year olds (60%) compared to the other age groups. COVID19 vaccine uptake was higher among pregnant women employed in businesses or institutions that required COVID19 vaccination (65%). However, Chi-square analysis confirmed no significant differences in vaccine uptake between the non-employed or those employed with or without work requirements for COVID19 vaccines.

### Associations Between Socio-demographic Characteristics and COVID19 Vaccination status

The results of the bivariate and multivariable analysis of socio-demographic characteristics associated with COVID19 vaccination are shown in Table 3. Compared to 25–29 years, the odds of COVID19 vaccination increased 3 times among women 30–39 years (OR: 3.08; 95% CI: 1.17–8.13, *P* =.02), and the results were statistically significant. The odds of COVID19 vaccination also increased 2.5 times among 18–24-year-olds and 3.33 times among older pregnant women (40–49 years), though the increase was not statistically significant.

**TABLE 3:** Logistic Regression: COVID-19 Vaccination Status and Socio-demographic Variables Among Pregnant Women in Kenya.

Characteristics	Unadjusted OR (95 %CI)	Adjusted OR (95 %CI)
Age		
18–24 years	2.5 (0.71–8.75)	3.96 (0.80–19.63)
25–29 years	Ref	Ref
30–39 years	3.08 (1.17–8.13)	3.81 (1.28–11.39)
40–49 years	3.33 (0.36–30.94)	–
Education level		
College/University	Ref	Ref
Secondary school	0.95 (0.38–2.39)	0.90 (0.31–2.64)
Primary school	0.17 (0.01–2.01)	0.17 (0.00–9.83)
Region		
Nairobi	Ref	Ref
Uasin Gishu	0.85 (0.37–1.94)	0.90 (0.32–2.54)
Insurance status		
Yes	Ref	Ref
No	0.81 (0.32–2.04)	0.75 (0.22–2.59)
Disability		
No	Ref	Ref
Yes	1.02 (0.30–3.47)	0.79 (0.17–3.61)
Comorbid condition		
No	Ref	Ref
Yes	1.71 (0.53–5.56)	1.45 (0.35–5.91)
Previous COVID-19 infection		
No	Ref	Ref
Yes	1.19 (0.51–2.76)	1.31 (0.50–3.45)
Workplace vaccine requirements		
No	Ref	Ref
Yes	2.81 (0.99–8.04)	4.65 (1.32–16.42)
Unemployed	0.99 (0.35–2.87)	1.24 (0.37–4.17)
Prior other vaccination		
No	Ref	Ref
Yes	1.03 (0.44–2.44)	1.01 (0.37–2.78)
Wearing a Mask		
Yes	Ref	Ref
No	1.38 (0.50–3.84)	3.13 (0.89–11.05)
Non-Health care workers	2.35 (1.55–3.58)	–

OR = odds ratio; CI = confidence interval; AOR = Adjusted odds ratio; Ref = reference category.

Higher education levels were associated with increased COVID19 vaccination, with pregnant women with college education 2.9 times more likely to receive COVID19 vaccination (OR: 2.9; 95% CI: 1.74 – 4.82, *P* =.001). The odds of COVID19 vaccination among pregnant women with primary and secondary education decreased by 83% and 5%, respectively, though not statistically significant. Compared to pregnant women who did not work in healthcare facilities, pregnant women working in healthcare settings had higher odds of COVID19 vaccination (OR 2.35; 95% CI: 1.55–3.58). However, the odds of vaccination by insurance status, disability, or prior COVID19 infections were not statistically significant.

Due to collinearity, the multivariable logistic regressions excluded the health worker variable and observations from pregnant women from the 40–49 age group. Adjusting for the level of education, insurance, comorbid conditions, and other variables, pregnant women in the 30–39 age group were 3.81 times more likely to have COVID19 vaccination than the reference group (AOR:3.81; 95% CI: 1.28–11.39, *P* =.017). Among the younger women (18–24 years), the adjusted odds were 3.96 times higher than the reference group, though the differences were not significant (AOR:3.89; 95% CI: 0.80–19.63).

The adjusted odds ratios for COVID19 vaccination decreased among pregnant women with secondary or primary level education, although the results were not statistically significant. Similarly, though having a prior COVID19 infection was associated with higher odds of COVID19 vaccination (AOR: 1.31; 95% CI:0.50–3.45), this finding was not significant in this study population. Pregnant women who reported wearing face masks had higher odds of COVID19 vaccination (AOR: 3.13, 95% CI: 0.89–11.04), but there was no evidence of association.

Regarding the workplace requirements for COVID19 vaccination, controlling for other variables, pregnant women who were employed in institutions or businesses that required COVID19 vaccination were 365% more likely to be vaccinated compared to those who worked in institutions that did not have COVID19 vaccine mandates (AOR: 4.65; 95% CI:1.32–16.42) (Table 3).

## DISCUSSION

Our study aimed to determine the prevalence of COVID19 vaccination and assess the relationship between COVID19 vaccination and socio-demographic characteristics among a sample of pregnant women seeking care at two national referral hospitals in Kenya. Results indicated a high prevalence of COVID19 vaccination (73%) in the study sample, with over 87% being fully vaccinated. Compared to the national data in Kenya, where less than 40% of the population is fully vaccinated against COVID19,^3,5,26^ the higher vaccination rates in our study could be attributed partly to efforts by the government of Kenya to scale up targeted vaccination among vulnerable populations, including pregnant women.^5^

By December 2022, the Ministry of Health in Kenya had approved six COVID-19 vaccines for use in Kenya, having received over 35 million doses of COVID-19 vaccines.^3,15,26^ In August 2021, the Kenya Obstetrical and and Gynaecological Society (KOGS) recommended COVID19 vaccination during pregnancy in consultation with healthcare providers.^27^ Our study reported a higher uptake of adenovirus vector vaccines (AstraZeneca and Johnson & Johnson) compared to uptake of the mRNA COVID19 vaccines (Pfizer and Moderna). Similar to other studies, the one dose schedule of the Johnson & Johnson vaccine was likely desirable for those with difficulty scheduling multiple appointments.^34^ Given that mRNA vaccines (Pfizer and Moderna) require ultracold storage,^40,41^ health facilities in Kenya with limited refrigeration equipment may have opted for adenovirus vector vaccines given the longer shelf life, thus making adenovirus vector COVID19 vaccines widely available compared to the mRNA COVID19 vaccines.^40^

Contrary to other studies where age and education were reported as significant predictors of COVID19 vaccination in the general population,^28,30,31^ our study had mixed mixed findings. The over representation of pregnant women with a college/university education could indicate that those with lower education levels may have been eliminated from the study if they could not read in English. Although a majority of pregnant women with either a secondary or college education had been vaccinated against COVID19, the insufficient evidence of an association between education and COVID19 vaccination in this sample is inconsistent with other studies where having a college education was associated with COVID19 vaccine acceptance in pregnancy,^42,43^ reflecting that many other information sources may have contributed to vaccine hesitancy among educated populations.

Some studies have reported that access to social media and a plethora of online information, “infodemic,” could have influenced vaccine decision-making, especially among well-educated individuals.^5,44,45^ One approach to reaching populations is to expand the dissemination of accurate health information beyond mainstream media (journals, TV, or radio) and to utilize social media and influencers on diverse platforms. Another strategy that has been used in other vaccine interventions involves incorporating vaccine recommendations in the health facility guidelines during routine check-ups to increase maternal vaccination.^46,47^

In our study, the statistically significant association between COVID19 vaccination uptake and older pregnant women (30–39 years) could suggest that older women felt more vulnerable to COVID19. The low rates of COVID19 vaccination among younger women (25–29 years) may have been due to messaging that primarily targeted older adults. Other studies have reported low vaccine rates among younger populations who felt less vulnerable to severe COVID19 infections.^28,42,40^

Similar to individuals in Cameroon, this present study did not show any relationship between medical comorbidities and COVID19 vaccination.^33^ Although other studies have also reported no significant associations between comorbid conditions during pregnancy (gestational diabetes mellitus or obesity) and COVID19 vaccination,^28,35^ the complexities between the perceived vulnerability to other infections that occur during pregnancy such as Tetanus, and vaccine hesitancy for COVID19 vaccines require further investigation.

This study was conducted in two national referral hospitals in Kenya. These facilities were chosen given the high volume of patients and the referrals for life-threatening health conditions. Our data revealed that only about one-fifth (1/5) had comorbid conditions during pregnancy, with about 81.0% vaccinated against COVID19 compared to 71.3% of those without comorbidities. The lack of significant results regarding COVID19 vaccination among pregnant individuals with comorbid conditions in this study population could suggest that pregnant women, regardless of their health conditions, may have a higher perceived vulnerability to severe COVID19 illness and thus opt for vaccination to protect themselves and their unborn children.

Policy responses to combat COVID19 through vaccination were extended beyond the national and county levels. While the government of Kenya initially used lockdowns and encouraged face coverings to mitigate COVID19,^48^ some institutions and businesses enforced COVID19 vaccination for their employees. Our findings suggested that workplace requirements for COVID19 were associated with COVID19 vaccination uptake and that all pregnant health workers in the study sample had received COVID19 vaccination. While vaccination mandates may have prompted COVID19 vaccination, it is unclear whether some pregnant women quit their jobs in search of other positions that did not have vaccine requirements or if some left formal employment during the COVID19 pandemic lockdowns and did not return to work. However, studies from other countries demonstrate that areas with COVID19 vaccination mandates/certifications had higher vaccination rates.^49,50^ Given the emerging evidence on COVID19 vaccination,^51^ it is important for governments to ensure that public institutions relay accurate messages and provide timely information to communities about maternal vaccinations.^52^ Future studies could explore the long-term influence of policies on vaccine decision-making among pregnant women in Kenya and whether behaviours change with subsequent pregnancies.

### Strengths and Limitations

This study utilized COVID19 vaccination questions from the NIS-ACM and the Omnibus surveys widely used across the US and adapted them to the Kenyan context. Results from this study will contribute to the ongoing research on demographic characteristics associated with COVID19 vaccination. By recruiting women from national hospitals, the study attempted to highlight the perspectives of pregnant women from diverse backgrounds and individuals likely to be high-risk patients in Kenya. This study is also timely as it addresses socio-demographic characteristics that could be targeted to improve other vaccination interventions during pregnancy.

Despite these strengths, several limitations should be noted. Since this was a cross-sectional study, we cannot establish temporality between socio-demographic characteristics and COVID19 vaccination status during pregnancy. However, results from this study provide a snapshot of the prevalence of COVID19 vaccination among pregnant women in Kenya. Information gathered from this study is critical in understanding socio-demographic determinants that influence COVID19 vaccination (or other vaccines) and the sub-populations to target to increase vaccine uptake. The use of WhatsApp for survey administration may have impacted generalizability because it excluded potential participants without access to the mobile application. Although studies show that about 97% of internet users in Kenya use WhatsApp as the primary application,^53,54^ it is unclear if this population felt comfortable using WhatsApp for other purposes rather than for communication and/or entertainment.

Study participants self-reported by responding to survey questions, which may have impacted responses due to recall bias. However, recollection bias was minimized by asking pregnant women to report the date (s) of vaccination and the vaccine brand received. Most clinics sent follow-up COVID19 vaccination schedules via text messages. The threat of social desirability or interview bias was also reduced by offering the survey online and ensuring participant confidentiality. Study subjects could terminate the survey whenever they felt uncomfortable and did not have to provide their name or contact information. The only contact information collected was used to confirm incentive payment using a mobile money transaction platform in Kenya.

## CONCLUSION

This study provided an estimated prevalence, and the demographic characteristics associated with the uptake of COVID19 vaccination among pregnant women in two national referral hospitals in Kenya. While this study showed higher rates of COVID19 vaccination receipt among pregnant women compared to the general rates in the Kenyan population, the variations of uptake by age group cohorts, education levels, and vaccine workplace requirements suggest a need for further and more robust research on COVID19 vaccination among pregnant women. The higher uptake of adenovirus vector COVID19 vaccines compared to mRNA vaccines could be due to the weak health system and health facilities in Kenya, which are not equipped with advanced technology or appliances to transport and store vaccines. Implementation of future health programs among pregnant women should consider factors within the local level to increase coverage and uptake of vaccination and other maternal preventative behaviours in Kenya and Sub-Saharan Africa.
